# Antimicrobial Protein Produced by Vaginal *Lactobacillus acidophilus* that inhibits *Gardnerella vaginalis*

**DOI:** 10.1155/S1064744901000060

**Published:** 2001

**Authors:** Alla A. Aroutcheva, Jose A. Simoes, Sebastian Faro

**Affiliations:** Department of Obstetrics and Gynecology Rush-Presbyterian-St. Luke's Medical Center 1653W. Congress Pkwy 720 Pav. Chicago IL 60612 USA

## Abstract

*Objective:* To isolate bacteriocin from a vaginal strain of *Lactobacillus acidophilus*.

*Methods: L. acidophilus* 160 was grown on two media. The first was MRS broth for 18 hours; the cells were harvested, washed, and placed into a chemically defined medium. The second medium resembled vaginal fluid minus protein. Bacteriocin was precipitated from both media using ammonium sulfate. The growth-inhibiting activity of bacteriocin was determined by a bioassay using nine different isolates of *Gardnerella vaginalis*.

*Results:* MRS broth is not a suitable medium for extracting bacteriocin, because it binds with Tween 80. Bacteriocin was isolated, without contaminating constituents, from chemically defined medium and identified as a single band by electrophoresis. Bacteriocin has a molecular weight of 3.8 kDa. All nine isolates of *Gardnerella* were inhibited by the bacteriocin isolated from *L. acidophilus* 160.

*Conclusions:* Bacteriocin produced by *L. acidophilus* 160 was isolated from the chemically defined medium (starvation medium) in a partially pure form. *L. acidophilus* 160 bacteriocin inhibited growth of all nine isolates of *Gardnerella vaginalis*.


*Infect Dis Obstet Gynecol 2001;9:33–39*

### Antimicrobial protein produced by vaginal *Lactobacillus acidophilus* that inhibits *Gardnerella vaginalis*

**Alla A. Aroutcheva, Jose A. Simoes and Sebastian Faro**

*Department of Obstetrics and Gynecology, Rush-Presbyterian-St. Luke's Medical Center, Chicago, IL*

***Objective:*** To isolate bacteriocin from a vaginal strain of *Lactobacillus acidophilus*.
***Methods:*** *L. acidophilus* 160 was grown on two media. The first was MRS broth for 18 hours; the cells were harvested, washed, and placed into a chemically defined medium. The second medium resembled vaginal fluid minus protein. Bacteriocin was precipitated from both media using ammonium sulfate. The growth-inhibiting activity of bacteriocin was determined by a bioassay using nine different isolates of *Gardnerella vaginalis*.
***Results:*** MRS broth is not a suitable medium for extracting bacteriocin, because it binds with Tween 80. Bacteriocin was isolated, without contaminating constituents, from chemically defined medium and identified as a single band by electrophoresis. Bacteriocin has a molecular weight of 3.8 kDa. All nine isolates of *Gardnerella* were inhibited by the bacteriocin isolated from *L. acidophilus* 160.
***Conclusions:*** Bacteriocin produced by *L. acidophilus* 160 was isolated from the chemically defined medium (starvation medium) in a partially pure form. *L. acidophilus* 160 bacteriocin inhibited growth of all nine isolates of *Gardnerella vaginalis*.

Key words: BACTERIOCINS; VAGINAL ECOSYSTEM

Lactobacilli, through the antagonistic interaction with pathogenic bacteria, maintain the vaginal ecosystem in a healthy state. Regulatory processes are carried out by species of *Lactobacillus* that produce antibacterial compounds, such as lactic and other organic acids, $H_2O_2$, and bacteriocins. Bacteriocins are biologically active, low-molecular-weight proteins or peptides that inhibit the growth of a variety of bacteria.

The bacteriocin activity includes other species of lactobacilli, as well as a variety of Gram-positive and Gram-negative aerobic, facultative, and obligate anaerobic bacteria. It is significant that one species of *Lactobacillus* will produce a bacteriocin that inhibits the growth of other lactobacilli. This may be one mechanism that allows *Lactobacillus* to dominate the ecosystem by suppressing not only other bacteria but also other lactobacilli. This in turn reduces competition within an ecosystem.

Several investigators have isolated and partially purified bacteriocin from different species of lactobacilli. Most of these investigations were conducted with nonhuman strains, predominantly isolated from food[1–9].

Human isolates of *Lactobacillus* species were found to have more antagonistic activity against other pathogenic microorganisms. A strain isolated from human feces produced a substance with potent inhibitory activity against a wide range of bacterial species. It inhibited anaerobic bacteria

Correspondence to: Alla A. Aroutcheva, Department of Obstetrics and Gynecology, Rush-Presbyterian-St. Luke's Medical Center, 1653W. Congress Pkwy, 720 Pav., Chicago, IL 60612, USA

(*Clostridium* spp., *Bacteroides* spp., *Bifidobacterium* spp.) and members of the family *Enterobacteriaceae*, *Pseudomonas* spp., *Staphylococcus* spp. and *Streptococcus* spp.; however, it did not inhibit other lactobacilli. The inhibitory activity occurred between pH 3 and pH 5 and was heat-stable[10].

*Lactobacillus gasseri,* which is considered the dominant species inhabiting the human intestine[11], was found to produce bacteriocin that exhibited a wide spectrum of bactericidal activity against enteric pathogens[12,13]. McGroarty and Reid[14] isolated antibacterial proteins from *Lactobacillus acidophilus* and *Lactobacillus casei* obtained from urethral specimens that were active against *Eshcerichia coli.* A heat-resistant peptide was extracted from a vaginal isolate, *Lactobacillus salivarius*, which inhibited growth of *Enterococcus faecalis*, *Enterococcus faecium*, and *Neisseria gonorrhoeae*[15].

Barefoot and Klaenhammer[16] found that 63% of *L. acidophilus* strains examined produced bacteriocin. Interest in *L. acidophilus* resulted from its ability to colonize the human intestinal tract[17]. Purified bacteriocin from intestinal *L. acidophilus* was found to be inhibitory to other lactobacilli as well as *E. faecalis*[18].

To date no report has been published on the recovery and isolation of inhibitory protein from the endogenous species of vaginal *Lactobacillus*, which inhibited the growth of *Gardnerella vaginalis*. This bacterium, *G. vaginalis*, appears to play a key role in disrupting the balance and equilibrium of the vaginal ecosystem. This results in an altered vaginal microflora, such as bacterial vaginosis.

The objective of this study was to develop a simple method for recovery and purification of the antimicrobial protein produced by endogenous vaginal *L. acidophilus*. Obtaining a pure bacteriocin will permit investigations leading to a better understanding of the interaction between the endogenous bacteria of the vagina.

### MATERIALS AND METHODS

### *Lactobacillus acidophilus* 160 and its cultivation conditions

*L. acidophilus* 160 was isolated from a patient with a healthy vaginal microflora, and the species was established using the MicroLog One system (Biolog Inc., Hayward, CA). The isolate was cultivated in MRS agar three times under anaerobic conditions at 37°C and tested for purity by using the Gram stain.

### Method A for isolating the antimicrobial active protein from MRS broth culture

*L. acidophilus* 160 was grown on MRS agar for 24 hours. Colonies were transferred into 30 ml of MRS broth and incubated in an anaerobic chamber (Forma Scientific, Marietta, OH) at 37°C for 18 hr. This culture was used to inoculate 2 liters of MRS broth and incubated in an anaerobic chamber at 37°C for 18 hours. The bacteria were harvested in the early exponential growth phase. Cells were removed from the broth culture by centrifugation at 1000 *g* for 25 min. The supernatant was passed through a Nalgene filter, pore size 0.45 μm (Nalge Nune Int., Rochester, NY). The pH was adjusted to 5.5 with 12% ammonium hydroxide.

Protein from the supernatant was precipitated using the fractional precipitation method of adding increasing concentrations of ammonium sulfate (20%, 30%, 40%, 50%, 60% and 80%). Precipitation was carried out in a cold room (5°C). After each precipitation, samples were centrifuged at 14 000 *g* for 25 min (5°C). This yielded three fractions; (1) surface layer, pellicle; (2) liquid layer; and (3) dark brown pellet. The first and third fractions were combined and dissolved in 5 ml phosphate-buffered saline (PBS), pH 7.2.

All protein samples were desalted by dialyzing at 5°C using dialysis tubing with MWCO 500 (Spectrum, Houston, TX) against 1 liter deionized water. The entire dialysis required four changes of deionized water over 3 days. The dialyzed protein solution was frozen at –70°C and lyophilized. Dried samples were stored at 5°C.

To separate protein agglomerated with Tween 80, the sample was defatted three times with chloroform/methanol in a ratio of 2:1[6]. The aqueous and chloroform/methanol layers were air dried, dissolved in a small amount of PBS, and tested for antibacterial activity.

### Method B for isolation of antimicrobial active protein from chemically defined media

After growing *L. acidophilus* 160 in 2 liters MRS broth in an anaerobic chamber for 18 hours at 37°C, culture broth was centrifuged and cells were separated from the supernatant. Removed cells were washed three times in PBS and transferred into 200 ml of the defined media (pH 6.0).

The defined media resembled vaginal secretions but lacked proteins and amino acids[19]. The medium formula for 1000 ml includes: NaCl 3.5 g, KCl 1.5 g, $K_2HPO_4$ 1.74 g, $KH_2PO_4$ 1.36 g, dextrose 10.8 g, cysteine HCl 0.5 g, glycogen 0.1% 1 ml, $MgS0_4$ 0.03% 0.3 ml, $NaHCO_3$ 0.004% 0.04 ml, vitamin K 10 mg, nicotinamide 1 mg, d-calcium pantothenate 1 mg, and biotin 0.01 mg.

Cells were incubated at 37°C for 18 hours in an anaerobic pouch (Oxoid Limited, Hampshire, England) with constant shaking. Cells were removed from the media by centrifugation. The supernatant was tested for hydrogen concentration, protein concentration, and bioactivity.

Ammonium sulfate at a concentration of 80% was used to precipitate protein from the defined media. The procedure was performed at 5°C by gradually adding a small amount of ammonium sulfate with continuously gentle stirring. After 3 hours, the suspension was centrifuged at 14 000 *g* for 25 min to yield two fractions, a pellet and a supernatant. Each fraction was dialyzed (MWCO 500) against deionized water, with four changes over 3 days. Both fractions were concentrated via lyophilization. Each fraction was tested for antibacterial activity. The supernatant fraction was diluted with PBS to obtain 30-fold concentration, and the pellet fraction was dissolved in 1 ml PBS. The concentration of protein in each fraction was determined using the Bradford method (Pierce, Rockford, IL) according to the Pierce manual.

### Demonstration of antimicrobial activity

Bacteriocin activity was determined by the agarwell diffusion method. A clinical strain of *G. vaginalis* was chosen as the target bacterium. A 0.5 McFarland suspension diluted 10 times with PBS was streaked onto the surface of the upper layer of a two-layered human blood agar (HBT; Becton Dickinson, Cockeysville, MD). After inoculation of the surface, agar was allowed to dry for 10 min. Eleven-millimeter wells were punched out in the upper layer of HBT agar and filled with the test sample. Plates were maintained on the benchtop for 1.5–2 hours for prediffusion and then incubated at 37°C in a 5% $CO_2$ atmosphere for 48 hours.

### SDS-PAGE

Ten to 20 % precast sodium dodecyl sulfate (SDS) polyacrylamide gel (PAGE; OWL, Portsmouth, NH) was used to separate proteins by molecular weights. Samples in a ratio of 1:2 were diluted with Laemmli sample buffer (Bio-Rad, Hercules, CA) containing 2-mercaptoethanol and were denatured by boiling for 2–5 min. Gel was run in 1× Tricin buffer 200 V for 30 min. Protein bands were visualized with Bio-Rad silver stain, according to the manufacturer's manual.

### RESULTS

### Method A

The cell-free crude supernatant of MRS broth had a pH of 4.0. The pH of the supernatant was adjusted to 5.5 by adding 12% ammonium hydroxide. This was done to negate the inhibitory effect of lactic acid as well as other organic acids produced by *L. acidophilus*. The supernatant was concentrated by lyophilization.

Bioassay for *G. vaginalis* was performed with the concentrated MRS crude supernatant (300 mg dissolved in 0.3 ml of PBS). MRS broth in the same concentration and Tween 80, 18 mg/ml (equal to the concentration in MRS broth) were used as controls. The MRS crude supernatant had a 17-mm zone of inhibition versus the control MRS broth at 15 mm and Tween 80 at 19 mm.

Bioassay of precipitated fractions demonstrated the highest activity in the fraction precipitated with 20% ammonium sulfate (zone of inhibition 7.5 mm). Increased concentrations of ammonium sulfate yielded decreasing amounts of protein as observed in a reduction in bioactivity, e.g., concentrations of ammonium sulfate at 30% (zone of inhibition was 5 mm), 40% (2.0 mm), and 50% (1.5 mm). No activity was observed at the 60% and 80% saturations.

After defatting with chloroform/methanol, the fractions obtained by precipitating with the 20% and 30% ammonium sulfate resulted in two layers, an upper aqueous layer and a lower brown layer containing chloroform/methanol. The upper fraction demonstrated activity, with a 3-mm zone of inhibition. The brown substance was biologically inactive.

When the 20% and 30% fractions were subjected to electrophoresis, a bow-shaped zone was recovered. The area was blurred and did not migrate as distinct bands. The MW of these bands was estimated to be between 8 and 10 kDa.

When a sample of Tween 80 was subjected to electrophoresis, the same four-shape band was obtained. However, the Tween 80 migrated to an area of high MW. The defatted sample contained six bands with high- to low-molecular-weight proteins (Figure 1).

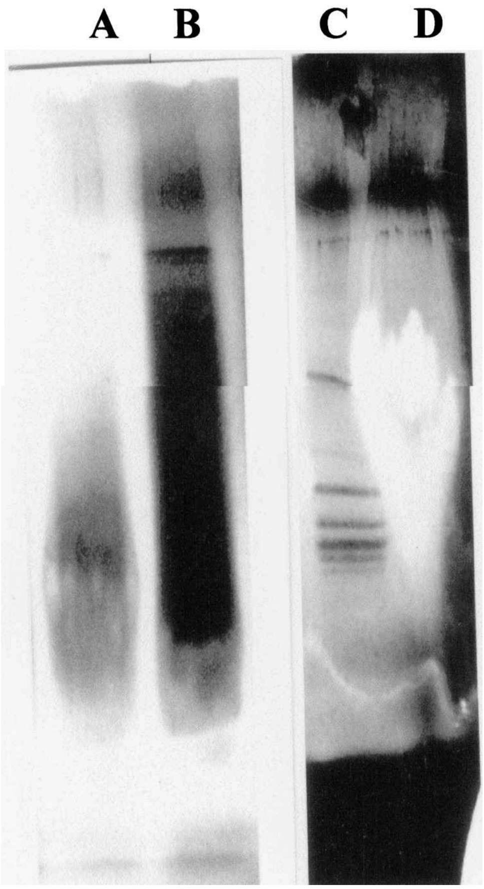

**Figure 1** SDS-PAGE of protein recovered from *L acidophilus* 160 MRS broth culture. **A:** Protein precipitated with the 20% ammonium sulfate. **B:** Protein precipitated with the 30% ammonium sulfate. **C:** Sample of protein defatted with chloroform/methanol. **D:** Tween 80

### Method B

In an attempt to obtain a pure protein and eliminate the effect of contaminating proteins and Tween 80, *L. acidophilus* 160 was grown in a defined medium for 18 hours then subsequently centrifuged to separate the cells from the medium. The supernatant without cells had a pH of 3.0 and a total concentration of protein of 11 250 μg.

The supernatant of defined medium was assayed for antimicrobial activity by adding 400 μl of nonconcentrated supernatant to the well. The sample developed a 5-mm zone of inhibition on HBT agar, with two zones; one zone (2 mm) was light brown with no bacterial growth, and the second zone (3 mm) had no color change or bacterial growth in the medium. The crude supernatant was lyophilized and concentrated 10× in PBS buffer. This sample was added to the well and tested against *G. vaginalis,* producing a zone of inhibition of 15 mm.

To exclude the possibility of lactic acid inhibiting the growth of *G. vaginalis*, lactic acid was added to the defined media to adjust pH to 3.0 and applied to a well for bioassay. The sample containing lactic acid had a zone of inhibition of 2 mm and was light brown.

SDS electrophoresis of the crude lyophilized supernatant produces a gel containing 10 bands of different-sized protein. The crude sample was subjected to 80% ammonium sulfate to precipitate the desired protein and reduce the total number of proteins in the crude sample. The mixture was centrifuged, and the pellet containing the desired protein was dissolved in PBS buffer. Both fractions were subjected to dialysis. Following dialysis, both fractions had a pH of 7.0. The concentration of protein in precipitated fraction was 150 μg, and in the supernatant 374.8 μg.

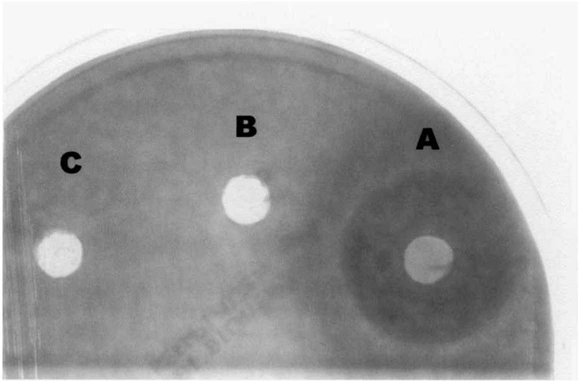

**Figure 2** Bioactivity of acidocin tested against *G. vaginalis*. **A:** Acidocin, obtained supernatant and not precipitated with 80% ammonium sulfate. **B.** Pellet fom precipitated with 80% ammonium sulfate protein. **C:** Control, defined media

Each fraction was assayed for bioactivity. The supernatant fraction was concentrated 30 times and exhibited a zone of inhibition that measured 8 mm (Figure 2). No change in blood agar color was observed, and the precipitate did not exhibit any bioactivity. Analysis of the supernatant sample on denaturing SDS-PAGE gels revealed a separate band, with MW 3.8 (Figure 3), which represents bacteriocin-acidocin.

### DISCUSSION

*Lactobacillus* plays a major role in maintaining a healthy vaginal ecosystem. This is accomplished by maintaining an acidic environment and suppressing the growth of other endogenous bacteria that constitute the vaginal microflora. Through the production of $H_2O_2$, organic acids and bacteriocins, lactobacilli prevent other bacteria, especially pathogenic bacteria, from overgrowing and thereby creating an altered vaginal microflora, e.g. bacterial vaginosis. Microbiologically, bacterial vaginosis is characterized by the replacement of *Lactobacillus* with *G. vaginalis* and anaerobes such as *Prevotella bivia* and *Peptostreptococcus* spp.

In a previous study, we showed that 78% of vaginal *Lactobacillus* spp. are able to inhibit growth of *G. vaginalis* (submitted for publication). The inhibition of growth effected by lactobacilli against *G. vaginalis* and other vaginal microflora was reported by Nagy, Skarin, and their colleagues[20,21].

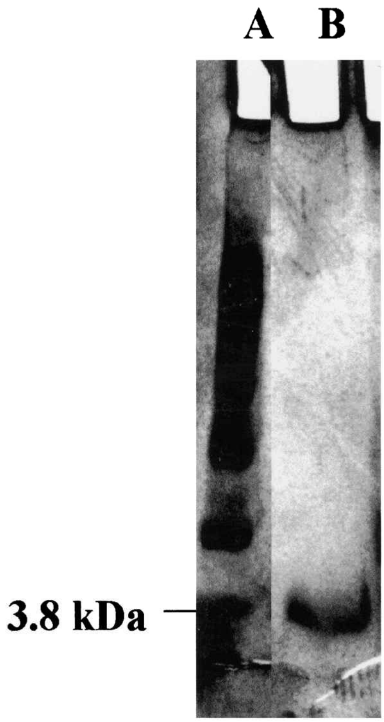

**Figure 3** SDS-PAGE of acidocin, MW 3.8 kDa. **A:** Molecular weight standard. **B:** Band of acidocin

In the present study, bacteriocin, a protein that inhibits growth of bacteria produced by vaginal *L. acidophilus*, was recovered and partially purified. The main difficulty in the isolation of bacteriocin using MRS medium is the presence of Tween 80. This compound is a nonionic surfactant, mostly composed of oleic acid that combined with bacteriocin and cannot easily be separated. It was shown that subsequently purified bacteriocin has an MW of 2.4 kDa; however, when it was combined with Tween 80, the MW was > 100 kDa[22].

In the present study, concentrated MRS broth inhibited growth of *G. vaginalis*. Further investigation revealed that this inhibition was caused by the Tween 80 constituent of MRS media. Strong bonds between Tween 80 and bacteriocin were proved by using the gradual ammonium sulfate precipitation method and SDS-PAGE. The active protein was extracted from MRS supernatant by precipitation with 20–30% ammonium sulfate.

Electrophoretic imaging of 20% and 30% precipitated protein revealed the same shape of the band as Tween 80. This proved to be a combination between Tween 80 and bacteriocin.

The band with a 'chicken footprint-like shape' was described by Kawai and colleagues[12] and was considered to be derived from Tween 80. Even intensive attempts to extract protein from Tween 80 with several organic solvents failed. Other investigators attempted to disrupt lactocin micelles from Tween 80 using methanol-chloroform and ethanol-diethyl ether extraction of fatty acids to separate the two proteins, 4.5 and 6 kDa[6].

In an attempt to improve bacteriocin isolation, several different media were used: MRS broth without proteins (meat extract); MRS broth without Tween 80[12]; and broth with casitone, yeast extract, amino acids and vitamins[23].

Media containing different combinations of glucose, amino acids and vitamins were studied by ten Brink and colleagues[22]. Glucose with amino acids, glucose with vitamins, and amino acids with vitamins did not support production of bacteriocin. A combination of all three agents – glucose, amino acids, and vitamins – supported production of bacteriocin-acidocin. These experiments demonstrated that growth is not necessary for acidocin production.

The present study confirmed the work of ten Brink and colleagues[22]. In that study, a medium composed of constituents resembling vaginal fluid was used[19]. The media contained salts, glucose and vitamins.

Inoculating this media with *L. acidophilus* 160 and incubating for 18 hours allowed for the excretion of organic acids and proteins. Bioassay of supernatant (pH 3.0) showed the presence of active substances in media that inhibited the growth of *G. vaginalis*. Using the defined media that had not been inoculated with *L. acidophilus* 160 and adjusting the pH to 3.0 with lactic acid demonstrated that the inhibition of *G. vaginalis* was caused by bacteriocin excreted by *L. accidophilus* 160. Bacteriocin of *L. acidophilus* 160 has an MW of 3.8 kDa.

This method is relatively simple and allows for recovery of biologically active protein, bacterocin or acidocin. The key step is taking bacteria in the log phase of growth and placing them in a defined medium that can be characterized as a starvation medium that stimulates the bacteria to secrete bacteriocin.

### REFERENCES

1. Larsen AG, Vogensen FK, Josephsen J. Antimicrobial activity of lactic acid bacteria isolated from sour doughs: purification and characterization of bavaricin A, a bacteriocin produced by *Lactobacillus bavaricus* MI401. *J Appl Bacteriol* 1993;75:113–22
2. Olasupo NA, Olukoya DK, Odunfa SA. Studies on bacteriocinogenic *Lactobacillus* isolates from selected Nigerian fermented foods. *J Basic Microbiol* 1995;35:319–24
3. Olasupo NA. Bacteriocins of *Lactobacillus plantarum* strains from fermented foods. *Fol Microbiol* 1996;41:130–6
4. Todorov S, Onno B, Sorokine O, *et al.* Detection and characterization of a novel antibacterial substance produced by *Lactobacillus plantarum* ST 31 isolated from sourdough. *Int J Food Microbiol* 1999; 48:167–77
5. Allison GE, Klaenhammer TR. Functional analysis of the gene encoding immunity to lactacin F, laf I, and its use as a *Lactobacillus*-specific, food-grade genetic marker. *Appl Environ Microbiol* 1996; 62:4450–60
6. Contreras BG, De Vuyst L, Devreese B, *et al.* Isolation, purification, and amino acid sequence of lactocin A, one of the two bacteriocins produced by *Lactobacillus* amylovorus LMG P-13139. *Appl Environ Microbiol* 1997;63:13–20
7. Coventry MJ, Muirhead K, Hickey MW. Partial characterisation of pediocin PO2 and comparison with nisin for biopreservation of meat products. *Int J Food Microbiol* 1995;26:133–45
8. Enan G, el-Essawy AA, Uyttendaele M, Debevere J. Antibacterial activity of *Lactobacillus plantarum* UGl isolated from dry sausage: characterization, production, and bactericidal action of plantaricin UGl. *Int J Food Microbiol* 1996;30:189–215
9. Ennahar S, Aoude-Werner D, Sorokine O, *et al.* Production of pediocin AcH by *Lactobacillus*

*plantarum* WHE 92 isolated from cheese. *Appl Environ Microbiol* 1996;62:4381–7
10. Silva M, Jacobus NV, Deneke C, Gorbach SL. Antimicrobial substance from a human *Lactobacillus* strain. *Antimicrob Agents Chemother* 1987;31: 1231–3
11. Fujisawa T, Benno Y, Yaeshima T, Mitsuoka T. Taxonomic study of the *Lactobacillus acidophilus* group, with recognition of *Lactobacillus gallinarum* sp. nov., and *Lactobacillus johnsonii* sp. nov., and synonymy of *Lactobacillus acidophilus* group A3 (Johnson *et al.* 1980) with the type strain of *Lactobacillus* amylovorus (Nakamura 1981). *Int J Syst Bacteriol* 1992;42:487–91
12. Kawai Y, Saito T, Toba T, *et al.* Isolation and characterization of a highly hydrophobic new bacteriocin (gassericin A) from *Lactobacillus gasseri* LA39. *Biosci Biotech Biochem* 1994;58:1218–21
13. Itoh T, Fujimoto Y, Kawai Y, *et al.* Inhibition of food-borne pathogenic bacteria by bacteriocins from *Lactobacillus gasseri. Lett Appl Microbiol* 1995;21: 137–41
14. McGroarty JA, Reid G. Detection of a *Lactobacillus* substance that inhibits *Escherichia coli. Can J Microbiol* 1988;34:974–8
15. Ocana VS, Pesce De Ruiz Holgado AA, Nader-Macias ME. Characterization of a bacteriocin-like substance produced by a vaginal *Lactobacillus salivarius* strain. *Appl Environ Microbiol* 1999;65: 5631–5
16. Barefoot SF, Klaenhammer TR. Detection and activity of lactacin B, a bacteriocin produced by *Lactobacillus acidophilus. Appl Environ Microbiol* 1983;45:1808–15
17. Kleeman EG, Klaenhammer TR. Adherence of *Lactobacillus* species to human fetal intestinal cells. *J Dairy Sci* 1982;65:2063–9
18. Muriana PM, Klaenhammer TR. Purification and partial characterization of lactacin F, a bacteriocin produced by *Lactobacillus acidophilus* 11088. *Appl Environ Microbiol* 1991;57:114–21
19. Geshnizgani AM, Onderdonk AB. Defined medium simulating genital tract secretions for growth of vaginal microflora. *J Clin Microbiol* 1992;30:1323–6
20. Nagy E, Petterson M, Mardh PA. Antibiosis between bacteria isolated from the vagina of women with and without signs of bacterial vaginosis. *Apmis* 1991;99:739–44
21. Skarin A, Sylwan J. Vaginal lactobacilli inhibiting growth of *Gardnerella vaginalis*, Mobiluncus and other bacterial species cultured from vaginal content of women with bacterial vaginosis. *Acta Pathol Microbiol Immunol Scand Sect B Microbiol* 1986,94:399–403
22. ten Brink B, Minekus M, van der Vossen JM, *et al.* Antimicrobial activity of lactobacilli: preliminary characterization and optimization of production of acidocin B, a novel bacteriocin produced by *Lactobacillus acidophilus* M46. *J Appl Bacteriol* 1994; 77:140–8
23. Joerger MC, Klaenhammer TR. Characterization and purification of helveticin J and evidence for a chromosomally determined bacteriocin produced by *Lactobacillus helveticus* 481. *J Bacteriol* 1986;167: 439–46

Received 10/30/00; Accepted 11/20/00
